# LncRNA MIR181A2HG inhibits keratinocytes proliferation through miR-223-3p/SOX6 axis

**DOI:** 10.18632/aging.205902

**Published:** 2024-06-06

**Authors:** Mingzhao Li, Mutian Niu, Xiaomei Fan, Fangru Chen, Hui Cao, Qingbo Liu, Shaoqin Gan, Pengpeng Yue, Jintao Gao

**Affiliations:** 1 School of Intelligent Medicine and Biotechnology, Guilin Medical University, Guilin 541199, Guangxi, P.R. China; 2 Key Laboratory of Biochemistry and Molecular Biology, Guilin Medical University, Education Department of Guangxi Zhuang Autonomous Region, Guilin 541199, Guangxi, P.R. China; 3 Department of Dermatology, Affiliated Hospital of Guilin Medical University, Guilin 541001, Guangxi, P.R. China; 4 Department of Dermatology, The Second Affiliated Hospital of Guilin Medical University, Guilin 541199, Guangxi, P.R. China

**Keywords:** psoriasis, MIR181A2HG, miR-223-3p, SOX6, keratinocytes

## Abstract

Background: Psoriasis is a complex and recurrent chronic inflammatory skin disease, and the abnormal proliferation of keratinocytes plays a crucial role in the pathogenesis of psoriasis. Long non-coding RNAs (lncRNAs) play an indispensable role in regulating cellular functions. This research aims to explore the potential impact of lncRNA MIR181A2HG on the regulation of keratinocyte proliferation.

Methods: The expression level of MIR181A2HG and the mRNA level of KRT6, KRT16, and SOX6 were assessed using qRT-PCR. The viability and proliferation of keratinocytes were evaluated using CCK-8 and EdU assays. Cell cycle analysis was performed using flow cytometry. Dual-luciferase reporter assays were applied to test the interaction among MIR181A2HG/miR-223-3p/SOX6. Protein level was detected by Western blotting analysis.

Results: The findings indicated that psoriasis lesions tissue exhibited lower levels of MIR181A2HG expression compared to normal tissue. The overexpression of MIR181A2HG resulted in the inhibition of HaCaT keratinocytes proliferation. The knockdown of MIR181A2HG promoted cell proliferation. The dual-luciferase reporter assay and rescue experiments provided evidence of the interaction among MIR181A2HG, SOX6, and miR-223-3p.

Conclusions: The lncRNA MIR181A2HG functions as a miR-223-3p sponge targeting SOX6 to regulate the proliferation of keratinocytes, which suggested that MIR181A2HG/miR-223-3p/SOX6 might be potential diagnostic and therapeutic targets for psoriasis.

## INTRODUCTION

Psoriasis, characterized by erythema and scales, is a chronic inflammatory skin disease, and the main pathological features are the overgrowth of keratinocytes in the lesion area of the patient and insufficient keratinization [1, 2]. The onset of psoriasis is associated with genetic, metabolic disorders, and immune disorders, and is prone to recurrence [3, 4]. Abnormal proliferation of keratinocytes has been proposed as a critical factor in the initiation, maintenance, and exacerbation of inflammation in psoriasis [5, 6]. However, the etiology of psoriasis remains unclear, and the pathogenesis is exceptionally complex, involving multiple factors such as immune dysregulation, inflammatory response, and abnormal cell proliferation during the occurrence and development of the disease [7]. Therefore, further investigation into the pathogenesis of psoriasis and the search for new therapeutic targets remains an urgent and important issue.

Long non-coding RNAs (lncRNAs) are RNA molecules with a transcription length of more than 200 nt that do not encode proteins. They are similar to mRNAs in some biological aspects but have some differences, displaying a more specific expression pattern and playing essential roles in maintaining cellular homeostasis and regulating cellular functions [8]. Some specific lncRNAs, such as MALAT-1 and PRINS, have been shown to influence keratinocyte proliferation and inflammation, thereby participating in the regulation of psoriasis pathogenesis [9, 10]. In previous studies, we also confirmed that knockdown of lncRNA MIR31HG inhibits proliferation in keratinocytes [11]. In recent years, microarrays and high-throughput sequencing have revealed a large number of differential lncRNAs in the skin lesions of psoriasis patients compared with normal skin, suggesting their potential involvement in psoriasis pathogenesis.

MIR181A2HG is a 617 nt lncRNA (NR_038975.1), and its potential impact on psoriasis remains unclear. In this study, we aimed to explore the expression of MIR181A2HG in psoriasis lesions and normal skin tissues, and investigate its potential roles in hyperproliferation of keratinocytes. We found that the low expression of MIR181A2HG in psoriatic lesions could promote the proliferation of keratinocytes through miR-223-3p/SOX6 axis.

## RESULTS

### MIR181A2HG was down-regulated in psoriatic skin lesions

**Figure 1:**
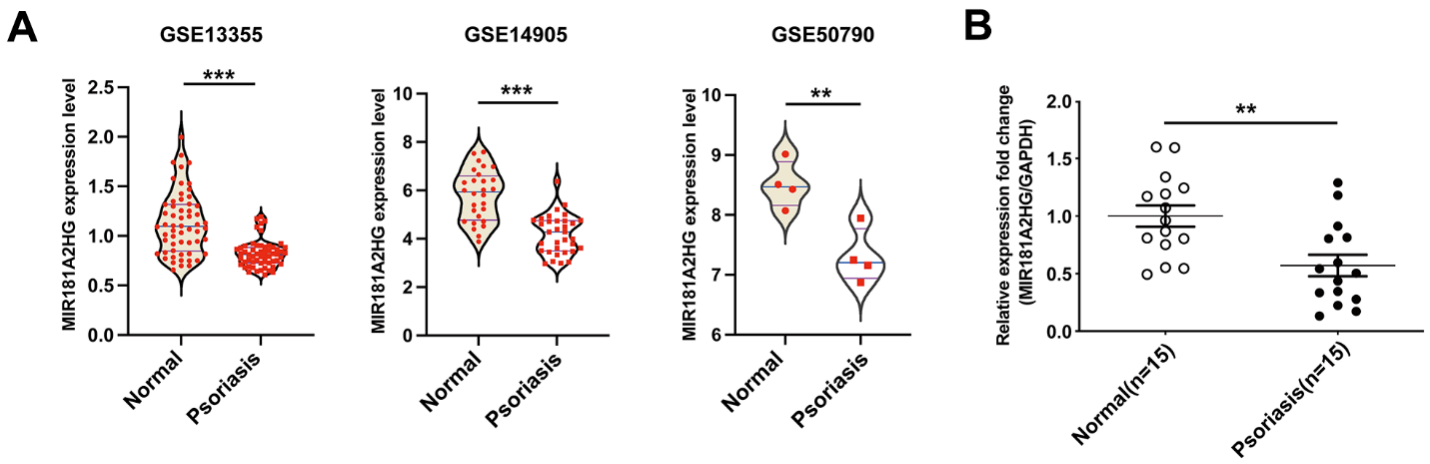
**MIR181A2HG was down-regulated in psoriatic skin lesions.** (**A**) The datasets GSE13355, GSE14905 and GSE50790 were downloaded to analyze the expression of MIR181A2HG in skin tissues. (**B**) qRT-PCR was performed to detect the expression of MIR181A2HG in skin tissues from psoriasis patients and normal individuals. **P<0.01, ***P<0.001.

To explore the expression level of lncRNA MIR181A2HG in psoriasis skin lesions, we downloaded the psoriasis-related GEO datasets (GSE13355, GSE14905, GSE50790) and conducted bioinformatics analysis. The results revealed a downregulation of MIR181A2HG expression in the skin lesion of psoriasis patients (Figure 1A). To confirm the expression of MIR181A2HG in clinical samples, the qRT-PCR was performed. The results showed that MIR181A2HG was lowly expressed in psoriatic skin tissues (Figure 1B), which was consistent with the results above.

### MIR181A2HG negatively regulated the proliferation of HaCaT keratinocytes

**Figure 2:**
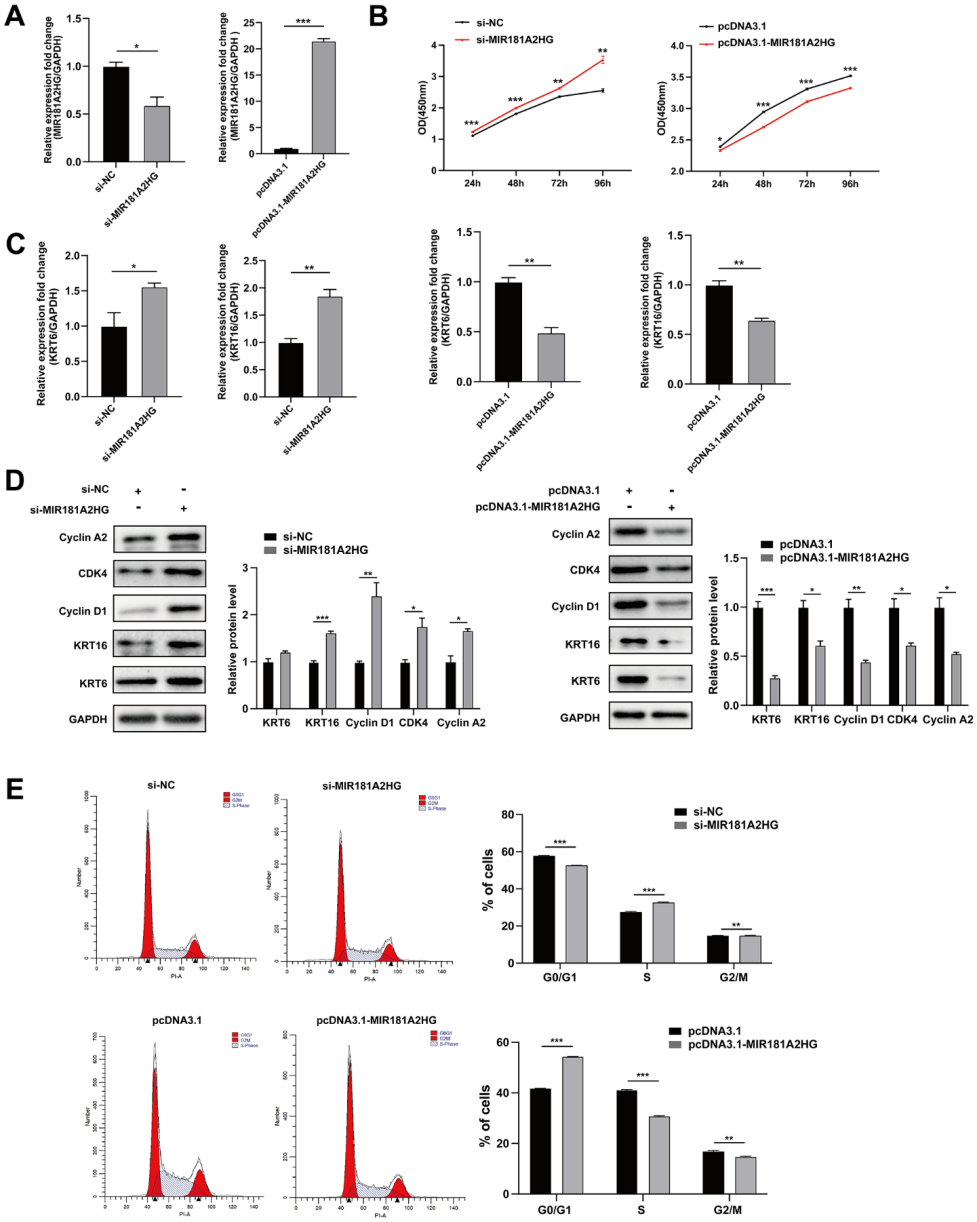
**MIR181A2HG negatively regulated keratinocytes proliferation.** (**A**) The efficiency of interference and overexpression of MIR181A2HG in HaCaT keratinocytes was detected. (**B**) The effects of interference or overexpression of MIR181A2HG on cell viability were assessed using CCK-8 kit. (**C**) HaCaT keratinocytes transfected for 48 hours were harvested and qRT-PCR was used to evaluate the effects of interference or overexpression of MIR181A2HG on KRT6 and KRT16 mRNA level. (**D**) Western blotting was applied to detect the effects on Cyclin A2, CDK4, Cyclin D1, KRT6 and KRT16 protein level. GAPDH served as an internal reference. (**E**) Flow cytometry was performed to analyze the effects on cell cycle distribution. *P<0.05, **P<0.01, ***P<0.001.

To investigate the potential role of MIR181A2HG in regulating the proliferation of keratinocytes, MIR181A2HG was overexpressed or knocked down in HaCaT keratinocytes and the effects on cell proliferation were evaluated. CCK-8 assay results revealed that MIR181A2HG knockdown did promote cell growth (Figure 2A, 2B). Keratin 6 (KRT6) and keratin 16 (KRT16) were considered as the hallmark of psoriatic keratinocytes hyperproliferation [12–14]. qRT-PCR and Western blotting results indicated that interfering with MIR181A2HG up-regulated the expression of KRT6 and KRT16 (Figure 2C, 2D). To investigate the possible reason why MIR181A2HG regulates the proliferation of keratinocytes, the cell cycle distribution was analyzed. The results showed that interference with MIR181A2HG led to an induction of cell transition from G0/G1 phase to S phase (Figure 2E). Moreover, Cyclin A2, Cyclin D1 and CDK4 were found to be upregulated with the MIR181A2HG interference (Figure 2D). The overexpression of MIR181A2HG exhibited an opposite trend (Figure 2). These results suggested that MIR181A2HG negatively regulated keratinocyte proliferation possibly associating with the disorder distribution of cell cycle.

### MIR181A2HG could sponge miR-223-3p

**Figure 3:**
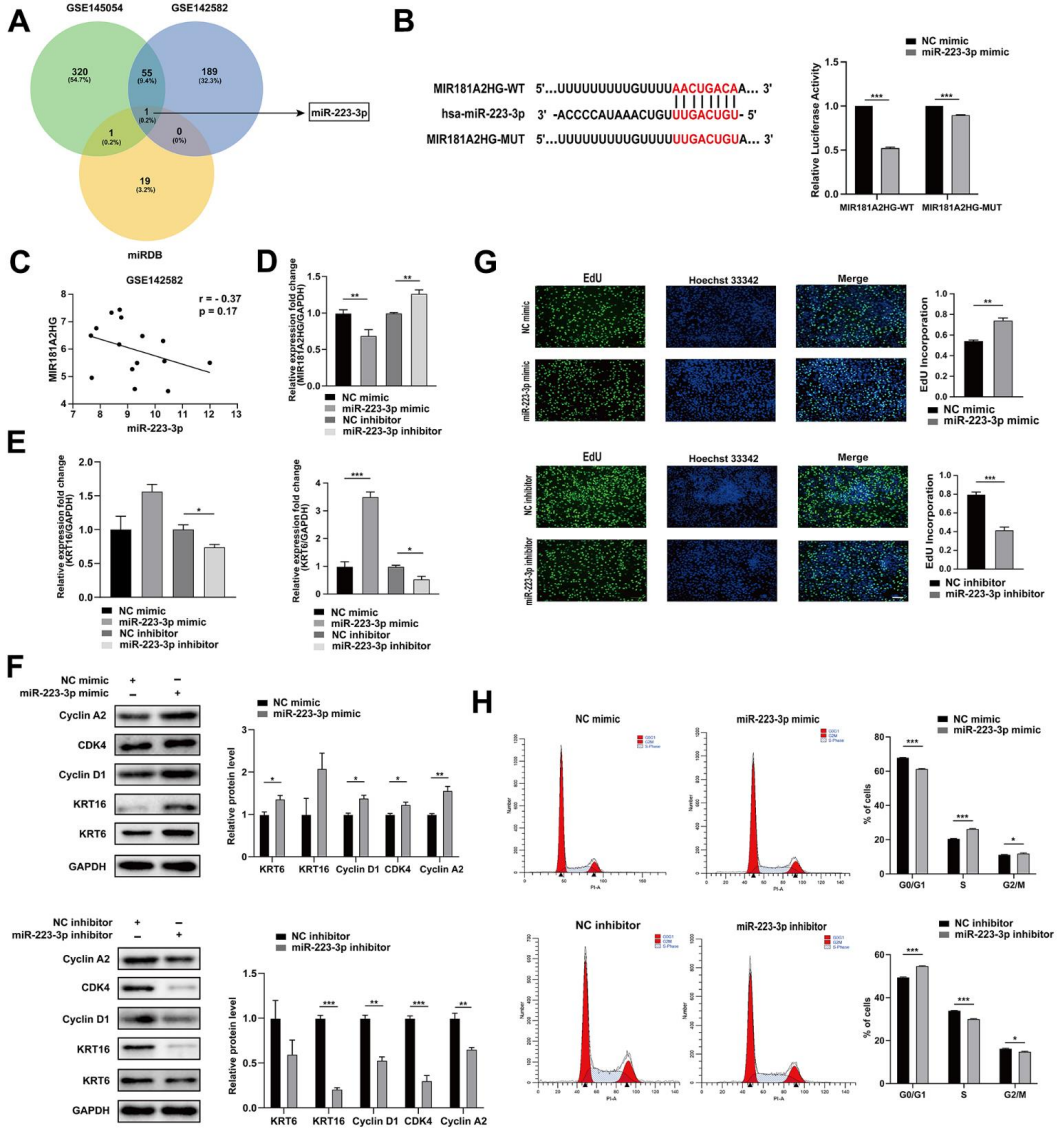
**MIR181A2HG sponged miR-223-3p.** (**A**) Venn diagram illustrates the intersection of upregulated miRNAs from GEO datasets (GSE145054 and GSE142582) and miRDB predicted targets of MIR181A2HG. (**B**) The binding site between MIR181A2HG and miR-223-3p. HaCaT keratinocytes were co-transfected with miR-223-3p/NC mimic and MIR181A2HG wide type (MIR181A2HG-WT)/MIR181A2HG mutant (MIR181A2HG-MUT) plasmid for 48 hours. Dual-luciferase reporter assay was performed to verify the interaction between MIR181A2HG and miR-223-3p. (**C**) Expression correlation of MIR181A2HG and miR-223-3p in dataset GSE142582. (**D**, **E**) qRT-PCR was used to detect the expression of MIR181A2HG, KRT6 and KRT16 in HaCaT keratinocytes with the transfection of miR-223-3p mimic/inhibitor. GAPDH served as an internal reference. (**F**) Western blotting was used to detect the protein level of Cyclin A2, CDK4, Cyclin D1, KRT6 and KRT16 in HaCaT keratinocytes with the transfection of miR-223-3p mimic/inhibitor. GAPDH served as an internal reference. (**G**) The rate of cell proliferation was assessed using EdU incorporation assays. Scale bar: 100 μm. (**H**) Flow cytometry was performed to analyze the effects on cell cycle distribution. *P<0.05, **P<0.01, ***P<0.001.

One of the mechanisms of action for lncRNAs involves their binding to miRNAs as competitive endogenous RNAs (ceRNAs), thus dynamically regulating the expression of downstream target mRNAs [15]. In order to search for miRNAs that MIR181A2HG may sponge, we extracted miRNAs that were upregulated from the GEO datasets (GSE142582 and GSE145054) and intersected with the miRNAs binding to MIR181A2HG which were predicted by the miRDB database (Figure 3A). The results indicate that miR-223-3p may interact with MIR181A2HG, and they are negatively correlated in GSE142582 (Figure 3C). The dual-luciferase reporter gene assay results show that miR-223-3p directly binds to MIR181A2HG (Figure 3B). The transfection of miR-223-3p mimic could decrease the expression of MIR181A2HG (Figure 3D), promote the expression of KRT6 and KRT16 (Figure 3E, 3F), improve the incorporation rate of EdU (Figure 3G). The modulation of miR-223-3p was found to affect Cyclin A2, Cyclin D1 and CDK4 expression (Figure 3F), and disturbed cell cycle distribution (Figure 3H). The opposite trend was found in the miR-223-3p inhibitor group (Figure 3D–3H). These results suggested that MIR181A2HG sponge miR-223-3p to regulate keratinocyte proliferation.

### miR-223-3p could target SOX6

**Figure 4:**
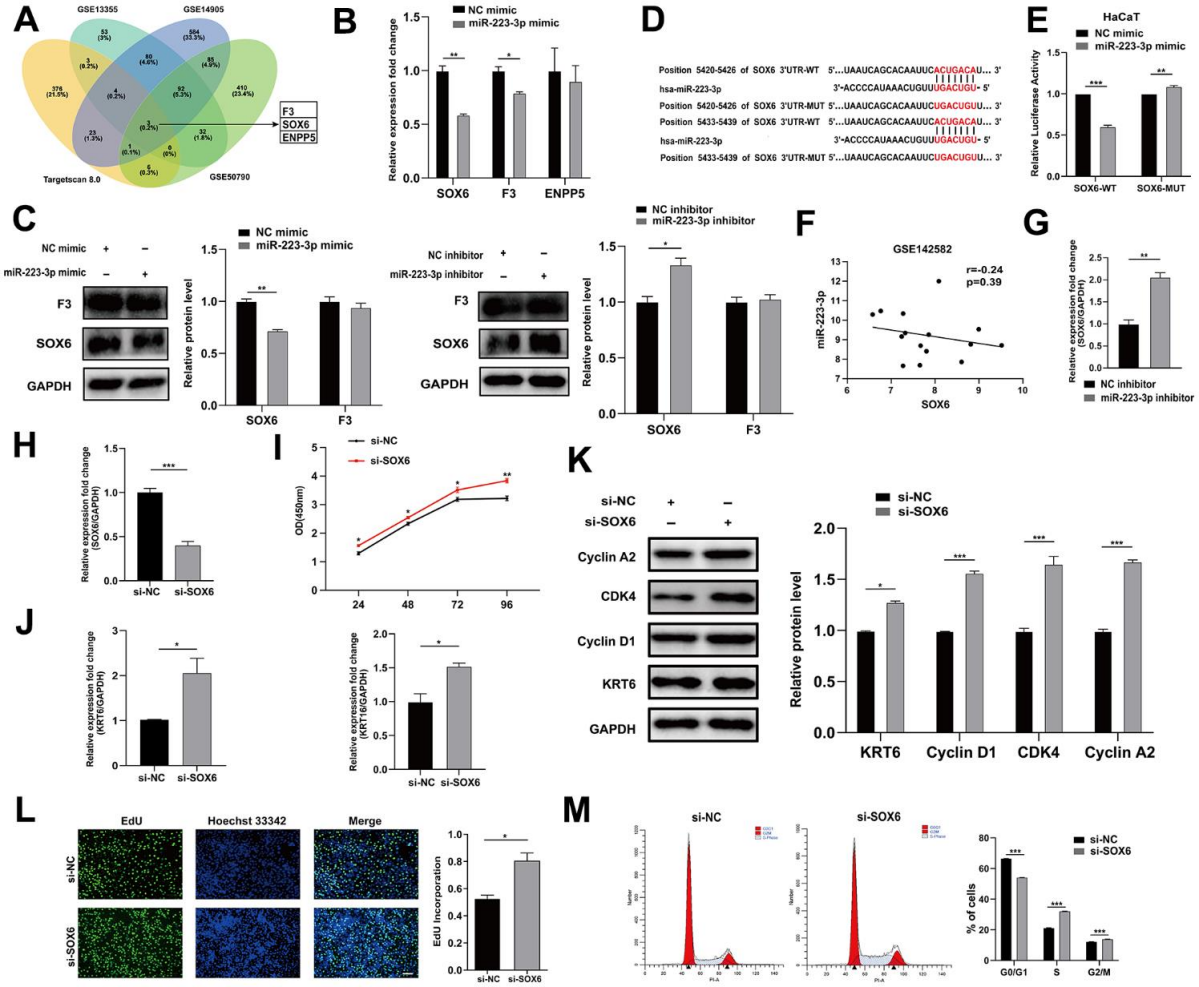
**miR-223-3p targeted SOX6.** (**A**) Venn diagram illustrates the intersection of downregulated genes from GEO datasets (GSE13355, GSE14905 and GSE50790) and TargetScan predicted targets of miR-223-3p. (**B**) qRT-PCR was performed to evaluate the effects of miR-223-3p mimic transfection on SOX6, F3 and ENPP5 mRNA level. GAPDH served as an internal reference. (**C**) Western blotting was used to detect the protein level of F3 and SOX6 in HaCaT keratinocytes with the transfection of miR-223-3p mimic/inhibitor. GAPDH served as an internal reference. (**D**) The binding site between miR-223-3p and SOX6. (**E**) HaCaT keratinocytes were co-transfected with miR-223-3p/NC mimic and SOX6 wide type (SOX6-WT)/SOX6 mutant (SOX6-MUT) plasmid for 48 hours. Dual-luciferase reporter assay was performed to verify the interaction between miR-223-3p and SOX6. (**F**) Expression correlation of miR-223-3p and SOX6 in dataset GSE142582. (**G**) qRT-PCR was used to detect the expression of SOX6 in HaCaT keratinocytes with the transfection of NC inhibitor/ miR-223-3p inhibitor. GAPDH served as an internal reference. (**H**) The efficiency of interference of si-SOX6 in HaCaT keratinocytes was detected. (**I**) The effects of SOX6 interference on cell viability were assessed using CCK-8 kit. (**J**) qRT-PCR was used to detect the expression of KRT6, KRT16 in HaCaT keratinocytes with the transfection of si-NC/si-SOX6. GAPDH served as an internal reference. (**K**) Western blotting was used to detect the protein level of Cyclin A2, CDK4, Cyclin D1 and KRT6 in HaCaT keratinocytes with the transfection of si-NC/si-SOX6. GAPDH served as an internal reference. (**L**) The rate of cell proliferation was assessed using EdU incorporation assays. Scale bar: 100 μm. (**M**) Flow cytometry was performed to analyze the effects on cell cycle distribution. *P<0.05, **P<0.01, ***P<0.001.

To identify the targets of miR-223-3p, we selected downregulated differential genes in GEO datasets (GSE13355, GSE14905, and GSE50790) and intersected with targets predicted by the Targetscan database, and F3, SOX6 and ENPP5 were screened (Figure 4A). After transfecting HaCaT keratinocytes with miR-223-3p mimics or inhibitors, and analyzing the mRNA and protein levels of SOX6, F3, and ENPP5, we found that SOX6 was significantly impacted by miR-223-3p (Figure 4B, 4C, 4G), and they are negatively correlated in GSE142582 (Figure 4F). The dual-luciferase reporter gene assays demonstrated that miR-223-3p directly binds to SOX6 (Figure 4D, 4E).

To investigate the potential role of SOX6 in regulating the proliferation of keratinocytes, HaCaT keratinocytes were transfected with si-SOX6 or si-NC and the effects on cell proliferation were evaluated (Figure 4H). Transfection of si-SOX6 promoted cell growth, increased the expression of KRT6 and KRT16, and improved the incorporation rate of EdU (Figure 4I, 4J, 4L). Interference with SOX6 also affected the expression of Cyclin A2, Cyclin D1 and CDK4, and disturbed cell cycle distribution (Figure 4K, 4M). In summary, these findings indicated that miR-223-3p could target SOX6 to regulate keratinocyte proliferation.

### MIR181A2HG affected expression of SOX6

**Figure 5:**
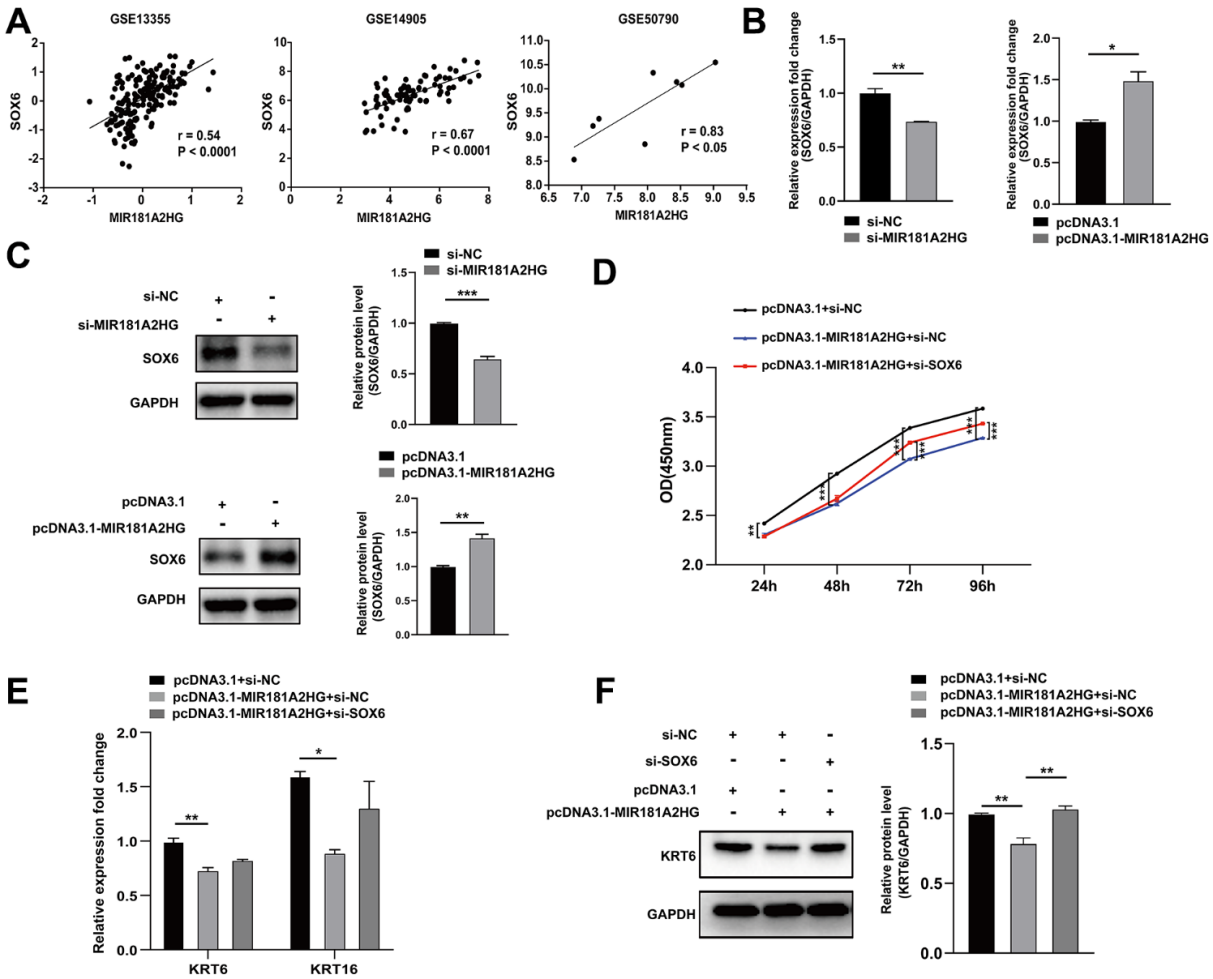
**The modulation of MIR181A2HG could affect SOX6 expression.** (**A**) Expression correlation of MIR181A2HG and SOX6 in datasets (GSE13355, GSE14905 and GSE50790). (**B**, **C**) HaCaT keratinocytes transfected for 48 hours were harvested and qRT-PCR/Western blotting was used to detect the expression of SOX6 in HaCaT keratinocytes with the transfection of si-MIR181A2HG/pcDNA3.1-MIR181A2HG. (**D**) CCK-8 kit was applied to detect the cell viability of HaCaT keratinocytes co-transfected with pcDNA3.1/pcDNA3.1-MIR181A2HG and si-NC/si-SOX6. (**E**, **F**) qRT-PCR and Western blotting were performed to detect the KRT6/KRT16 level in HaCaT keratinocytes co-transfected with pcDNA3.1/pcDNA3.1-MIR181A2HG and si-NC/si-SOX6. GAPDH served as an internal reference. *P<0.05, **P<0.01, ***P<0.001.

MIR181A2HG was found to exhibit a positive correlation with SOX6 in GEO datasets (Figure 5A). To determine whether MIR181A2HG affects the expression of SOX6, si-MIR181A2HG/pcDNA3.1-MIR181A2HG was transfected into HaCaT keratinocytes, and the expression of SOX6 was analyzed. The decrease in both mRNA and protein levels of SOX6 in response to MIR181A2HG knockdown was observed. The group with overexpressed MIR181A2HG exhibited the contrary trend (Figure 5B, 5C). To confirm whether MIR181A2HG mediated inhibition of keratinocytes proliferation was indeed achieved through SOX6, pcDNA3.1-MIR181A2HG plasmid, si-SOX6, or empty control were transfected into HaCaT keratinocytes. pcDNA3.1-MIR181A2HG plasmid transfection inhibited cell growth and decreased the expression of KRT6 and KRT16, which could be restored with the co-transfection of si-SOX6 (Figure 5D–5F).

### MIR181A2HG regulated keratinocytes proliferation via miR-223-3p/SOX6 axis

**Figure 6:**
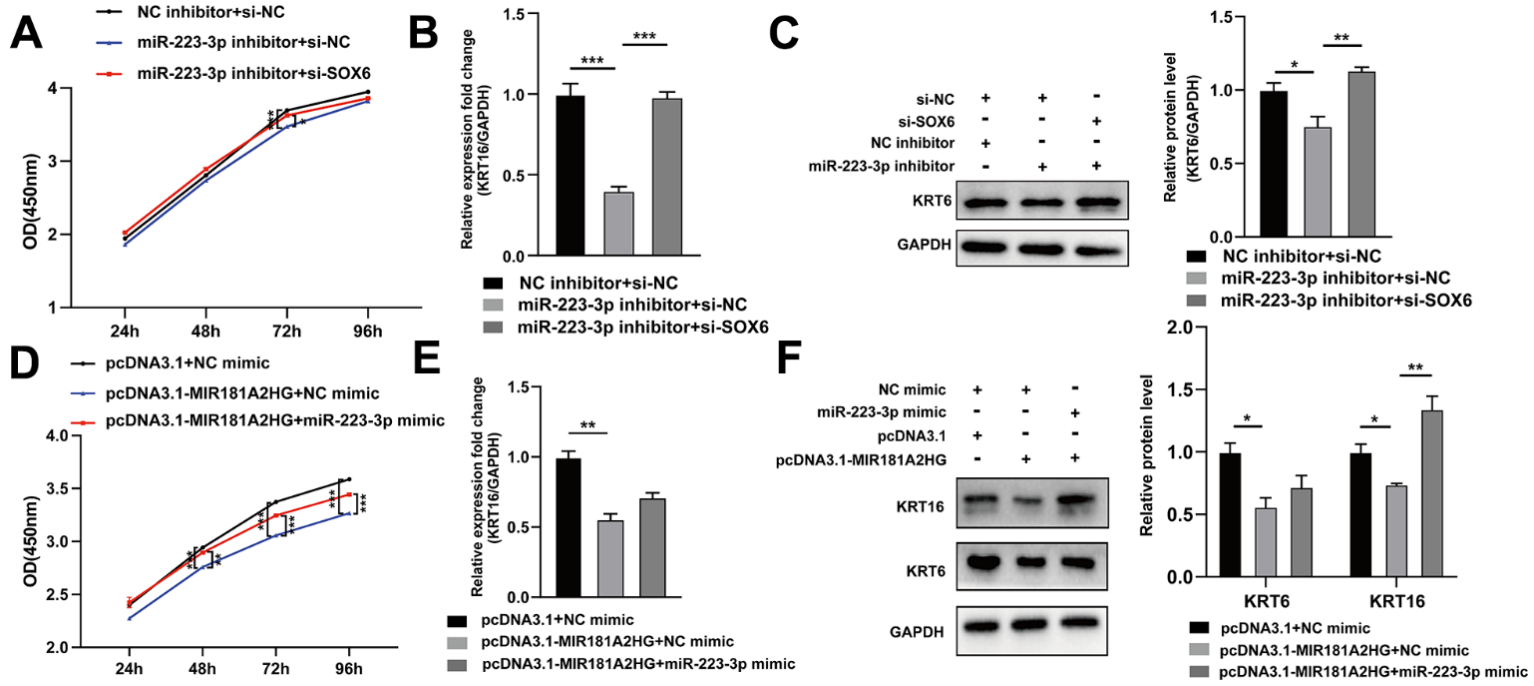
**The MIR181A2HG/miR-223-3p/SOX6 axis regulated the proliferation of HaCaT keratinocytes.** (**A**) CCK-8 kit was applied to detect the cell viability of HaCaT keratinocytes co-transfected with NC inhibitor/miR-223-3p inhibitor and si-NC/si-SOX6. (**B**, **C**) qRT-PCR and Western blotting were performed to detect the KRT6/KRT16 level in HaCaT keratinocytes co-transfected with NC inhibitor/miR-223-3p inhibitor and si-NC/si-SOX6. (**D**) CCK-8 kit was applied to detect the cell viability of HaCaT keratinocytes co-transfected with pcDNA3.1/pcDNA3.1-MIR181A2HG and NC mimic/miR-223-3p mimic. (**E**, **F**) qRT-PCR and Western blotting were performed to detect the KRT6/KRT16 level in HaCaT keratinocytes co-transfected with pcDNA3.1/pcDNA3.1-MIR181A2HG and NC mimic/miR-223-3p mimic. GAPDH served as an internal reference. *P<0.05, **P<0.01, ***P<0.001.

In order to further determine the relationship among MIR181A2HG, miR-223-3p, and SOX6, HaCaT keratinocytes were transfected with miR-223-3p inhibitor, si-SOX6 (or pcDNA3.1-MIR181A2HG plasmid, miR-223-3p mimic), or empty control. Transfection of miR-223-3p inhibitor inhibited cell growth and decreased the expression of KRT6 and KRT16, which could be restored with the co-transfection of si-SOX6 (Figure 6A–6C). Similarly, transfection of the pcDNA3.1-MIR181A2HG plasmid alone impeded the growth of keratinocytes, decreased the expression of KRT6 and KRT16, whereas co-transfection of the pcDNA3.1-MIR181A2HG plasmid with miR-223-3p mimics relieved this effect (Figure 6D–6F). These results suggested that MIR181A2HG could regulate keratinocytes proliferation via miR-223-3p/SOX6 axis.

## DISCUSSION

Psoriasis is one of the vital autoimmune diseases at present, which significantly impacts the physical and mental well-being, as well as the quality of life, of affected individuals [16]. In addition, psoriasis is closely associated with complications such as psoriatic arthritis, cardiovascular disease, and metabolic syndrome [17–19]. It is thought that the dynamic interaction between keratinocytes and immune cells could contribute to the occurrence and development of psoriasis [20]. Previous studies have provided evidences supporting the involvement of lncRNAs in the modulation of keratinocytes, thus influencing the pathogenesis of psoriasis. For instance, Jia et al. demonstrated that overexpression of the lncRNA MEG3 inhibits the inflammatory response in HaCaT and NHEK cells [21]. LncRNA AGXT2L1-2 was found to facilitate keratinocytes proliferation and inhibits apoptosis by interacting with estrogen-related receptor alpha in psoriasis [22]. In the current study, we demonstrated that MIR181A2HG was lowly expressed in the skin lesion of psoriasis patients. *In vitro* studies showed that interference with MIR181A2HG promoted abnormal proliferation of keratinocytes and overexpression of MIR181A2HG yielded contrasting outcomes.

It has been reported that lncRNA mediated ceRNA regulatory networks play a crucial role in psoriasis. For instance, lncRNA H19 promotes abnormal proliferation of keratinocytes and skin inflammatory responses by adsorbing miR-766-3p, regulating the expression of S1PR3 [23]. LncRNA AGAP2-AS1 is up-regulated in the lesion area of psoriasis patients and regulates keratinocytes via the miR-424-5p/AKT3 axis [24]. In this study, we successfully identified the binding sites of MIR181A2HG/miR-223-3p and miR-223-3p/SOX6, which were verified via dual-luciferase reporter assay. Furthermore, we further verified the connection among MIR181A2HG, miR-223-3p and SOX6 using rescue experiments. Based on these findings, we proposed a molecular mechanism involving the MIR181A2HG/miR-223-3p/SOX6 axis in regulating abnormal psoriatic keratinocyte proliferation.

MicroRNAs (miRNAs) are a class of conserved non-coding RNAs with a length of approximately 18-25 nt. Numerous dysregulated miRNAs have been reported to participate in regulating the function of keratinocytes in psoriasis. They could significantly impact entire cellular pathways through their interactions with target genes [25, 26]. miR-223-3p, as an important regulatory miRNA, has been found to be widely involved in the regulation of cell proliferation, apoptosis, and inflammatory response, thereby participating in the occurrence and development of diseases [27–29]. miR-223 (miR-223-3p) has been found to be highly expressed in the blood, plasma, PBMCs and skin lesions of psoriasis patients, playing an important role in inflammatory response activation, T cell differentiation, and regulation of keratinocyte proliferation [30–33]. It is considered a novel biomarker for disease activity in psoriasis. In this study, we found that miR-223-3p functions as a target miRNA sponged by MIR181A2HG. miR-223-3p was found to positively regulate keratinocyte proliferation, which may result from its binding to SOX6. SOX6 (SRY-Box Transcription Factor 6) is an important transcription factor that participates in the regulation of cellular functions and exerts an impact on the occurrence and development of various diseases [34, 35]. SOX6 is abnormally expressed in the epidermis of atopic dermatitis patients, where it impairs skin barrier development through suppressing epidermal differentiation and epigenetically silences critical genes involved in keratinocyte differentiation by recruiting SMARCA complex components [36]. In this study, we observed that SOX6 serves as a target of miR-223-3p, which is involved in negatively regulating the proliferation of keratinocytes.

## CONCLUSIONS

The present study demonstrated that lncRNA MIR181A2HG plays a key role in the proliferation of keratinocytes by regulating miR-223-3p targeting SOX6, which suggests that MIR181A2HG may be a potential diagnostic and therapeutic target for psoriasis. However, the upstream molecular mechanisms for MIR181A2HG regulation, and the downstream mechanism mediated by SOX6 are worth further exploration.

## MATERIALS AND METHODS

### Tissue sample collection

A total of 15 patients with psoriasis (8 males, 7 females, mean age 55.6 years) who had not received any treatment for nearly 4 weeks were selected for punch biopsy (4 mm). Healthy skin samples were obtained from individuals undergoing plastic surgery. All samples were frozen using liquid nitrogen. Prior to the study, informed consent was obtained from all patients, and the Ethics Committee of Guilin Medical University approved the study (GYLL2018060, 3/2/2018, Guilin, Guangxi, China).

### Cell culture

The HaCaT keratinocytes were cultured in an incubator at 37° C with 5% CO_2_. Cell passaging was performed every two to three days. Briefly, the cells were washed with PBS, digested with trypsin, collected, and transferred to a culture dish containing fresh medium supplemented with 10% serum.

### Plasmid construction, siRNA synthesis and cell transfection

Recombinant plasmids expressing lncRNA MIR181A2HG were constructed based on pcDNA3.1 backbone and transfected into HaCaT keratinocytes according to jetPRIME reagent instructions (Polyplus, France). Specific small interfering RNA (siRNA) and control siRNA (siNC) were synthesized by Gene Pharma (Shanghai, China). The sequence of si-MIR181A2HG was as follows: 5’-AGGUAGAUUCUGCAUCCACTT-3’. The sequence of si-SOX6 was as follows: 5’-GGGAAAGUCAAAUGAAGAUTT-3’.

### RNA extraction and quantitative real-time PCR analysis

The logarithmic growth phase HaCaT keratinocytes were seeded in a 12-well plate, and cells were harvested 48 hours after transfection. Trizol (Invitrogen, USA) was used to extract RNA from tissue samples or cell according to standard protocols. First strand of cDNA was synthesized with 2 μg of total RNA using RevertAid First Strand cDNA Synthesis Kit (Thermo Fisher Scientific, USA). Quantitative PCR (qPCR) was executed using the SYBR Green qPCR Master Mix kit (GK10002, GLPBIO, USA). The quantitative PCR was carried out in the CFX96 Touch™ Real-Time PCR Detection System (Bio-Rad, USA). Thermocycling conditions were as follows: 95° C for 5 min, followed by 40 cycles at 95° C for 5 s, 60° C for 10 s, and 72° C for 10 s. The expression levels were normalized to GAPDH and the relative expression was calculated by 2^-ΔΔCt^. Primer sequences were as follows:

MIR181A2HG, 5’-GTCGTTGCTGCTTTCTCCCA-3’ (forward) and

5’-ACGGATCGAGAGCCTGTTAC-3’ (reverse), 231 bp (product size);

KRT6, 5’-GGGTTTCAGTGCCAACTCAGCCAGGC-3’ (forward) and

5’-CCATACAGACTGCGG-3’ (reverse), 141 bp (product size);

KRT16, 5’-ATTCTTCCCGCGAGGTCTTC-3’ (forward) and

5’-CTGTGGTAGAGGCAGCTCAG-3’ (reverse), 132 bp (product size);

SOX6, 5’-CTCTTGTTCAGTCCGAGTCA-3’ (forward) and

5’-GGACAGCGTTCTGTCATCTC-3’ (reverse), 238 bp (product size);

ENPP5, 5’-AAGCGCTTTCCTACTCATTACA-3’ (forward) and

5’-GTCATCAGGGTCTTCCCAATAG-3’ (reverse), 135 bp (product size);

F3, 5’-CGACGAGATTGTGAAGGATGT-3’ (forward) and

5’-CGAGGTTTGTCTCCAGGTAAG-3’ (reverse), 146 bp (product size);

GAPDH, 5’-CACATGGCCTCCAAGGAGTAA-3’ (forward) and

5’-TGAGGGTCTCTCTCTTCCTCTTGT-3’ (reverse), 75 bp (product size).

### Cell counting kit-8 (CCK-8) assay

HaCaT keratinocytes were seeded into 96-well plates and cultured for 1, 2, 3, and 4 days. 10 μL of CCK-8 kit was added daily (Beyotime, Beijing, China), and the cells were incubated for 2 hours. The absorbance at 450 nm was measured using a multiskan microplate reader (Thermo Fisher Scientific, USA).

### Flow cytometry analysis

HaCaT keratinocytes were collected after 48 hours of transfection. Then, cells were washed twice with PBS buffer, fixed with 70% ethanol, and stored overnight at 4° C. After staining with 500 μl of staining buffer, 25 μl of propidium iodide staining solution, and 10 μl of RNaseA (Li Kee Bio, Shanghai, China) were added and incubated for 30 min. Cell cycle analysis was performed using FACSAria III flow cytometry (BD Biosciences, USA).

### Dual-luciferase reporter assay

The recombinant psiCHECK2.0-MIR181A2HG-MUT (mutant) and psiCHECK2.0-MIR181A2HG-WT (wild-type) plasmids, as well as the psiCHECK2.0-SOX6-MUT (mutant) and psiCHECK2.0-SOX6-WT (wild-type) plasmids, were constructed. HaCaT keratinocytes were co-transfected with psiCHECK2.0-MIR181A2HG-WT/psiCHECK2.0-MIR181A2HG-MUT (1 μg per well) and miR-223-3p mimic (100 nM). Similarly, HaCaT keratinocytes were co-transfected with psiCHECK2.0-SOX6-WT/psiCHECK2.0-SOX6-MUT (1 μg per well) and miR-223-3p mimic (100 nM). The dual-luciferase reporter assay system (Promega, USA) was used to detect dual-luciferase activity after 48 hours.

### Western blotting

The cells were lysed by adding RIPA lysis buffer (Beyotime) supplemented with protease inhibitors. Following that, the same amount of protein (40 μg per well) was used to perform sulfate-polyacrylamide gel electrophoresis (SDS-PAGE) and transferred to a polyvinylidene fluoride (PVDF) membrane (Millipore, USA) after electrophoresis. Subsequently, the PVDF membrane was blocked at room temperature in TBST solution containing 5% milk for 2 hours. Then, the PVDF membrane was incubated overnight with primary antibodies. The rabbit monoclonal antibodies [Anti-Cyclin A2 (ab32386, 1:10000), Anti-CDK4 (ab108357, 1:10000), Anti-Cyclin D1 (ab134175, 1:10000), Anti-KRT16 (ab181055, 1:10000)] were purchased from Abcam (UK), and mouse monoclonal antibodies [Anti-KRT6 (E2250041, 1:1000), Anti-SOX6 (E1A10565, 1:1000), Anti-GAPDH (E12-042, 1:1000)] were purchased from EnoGene (China). Mouse monoclonal antibodies [Anti-F3 (TA324022S, 1:1000)] were purchased from ORIGENE (China).

### 5-ethynyl-2’-deoxyuridine (EdU) assay

The EdU assay was performed using the EdU staining kit (Beyotime). HaCaT keratinocytes were seeded into 6-well plates, transfected for 48 hours. Subsequently, cells are labeled with EdU and subjected to subsequent experimental operations including cell fixation, cell washing, and cell permeability. The click reaction solution was added and incubated for 30 minutes at room temperature in the dark. Hoechst 33342 staining solution was prepared for nuclear staining and incubated for 10 minutes in the dark. Fluorescence microscopy was used to detect the fluorescence. EdU incorporation rate was evaluated using ImageJ software.

### Bioinformatics analysis and target prediction

The datasets GSE13355 (58 psoriatic patients and 64 normal healthy controls), GSE14905 (21 normal healthy donors and 28 psoriasis patients), GSE50790 (4 lesional and uninvolved skin samples obtained from patients with stable chronic plaque psoriasis), GSE142582 (5 adult psoriasis patients and 5 control skin samples), and GSE145054 (4 skin biopsies of lesional psoriatic skin and 4 healthy control skin samples) were obtained from the Gene Expression Omnibus (GEO) database (https://www.ncbi.nlm.nih.gov/geo/). Series Matrix File(s) and platform annotation files were downloaded for matching probes and extracting data. Differentially expressed genes (DEGs) were obtained by the “limma” package of R software. The up-regulated miRNAs in psoriatic lesions were screened (log2 (FC) >1, FDR <0.05) and the target miRNAs of MIR181A2HG were screened in combination with the miRDB database (https://mirdb.org/). In addition, the down-regulated coding genes in psoriasis lesions were analyzed (log2 (FC)<-1, FDR<0.05) and combined with TargetScan databases (https://www.targetscan.org/vert_80/) to screen target genes of miR-223-3p.

### Statistical analysis

Data are presented as means ± standard errors of at least three independent experiments. Statistical analysis was conducted using GraphPad Prism 9.0 statistical software. Student’s *t*-test was used for comparison, and a *p*-value of less than 0.05 was deemed statistically significant.
